# Immunosuppressant-Associated Neurotoxicity Responding to Olanzapine

**DOI:** 10.1155/2014/250472

**Published:** 2014-07-09

**Authors:** James A. Bourgeois, Ana Hategan

**Affiliations:** ^1^Department of Psychiatry, Langley Porter Psychiatric Institute, Consultation-Liaison Service, University of California San Francisco Medical Center, 401 Parnassus Avenue, San Francisco, CA 94143, USA; ^2^Department of Psychiatry and Behavioural Neurosciences, Michael G. DeGroote School of Medicine, Faculty of Health Sciences, McMaster University, St. Joseph's Healthcare Hamilton, 100 West 5th Street, Hamilton, ON, Canada L8N 3K7

## Abstract

Immunosuppressants, particularly tacrolimus, can induce neurotoxicity in solid organ transplantation cases. A lower clinical threshold to switch from tacrolimus to another immunosuppressant agent has been a common approach to reverse this neurotoxicity. However, immunosuppressant switch may place the graft at risk, and, in some cases, continuation of the same treatment protocol may be necessary. We report a case of immunosuppressant-associated neurotoxicity with prominent neuropsychiatric manifestation and describe psychiatric intervention with olanzapine that led to clinical improvement while continuing tacrolimus maintenance.

## 1. Introduction

Neurotoxicity is a concerning complication of immunosuppressive therapy, manifesting as various psychiatric and/or neurological symptoms [1, 2]. Tacrolimus, a calcineurin inhibitor, has been one of the cornerstones of immunosuppressive protocols in organ transplantation [1]. In order to reduce the incidence of posttransplant complications (including rejection of the graft), current strategies that are used for induction and maintenance therapy include the concomitant use of mycophenolate and corticosteroids, which may also come with their own set of side effects.

Emergence of neurotoxicity due to immunosuppressants, particularly tacrolimus, was reported as being dose dependent; tacrolimus dose reduction or switch to an alternative immunosuppressive regimen has resulted in clinical improvement [2, 3]. However, the immunosuppressant switch can lead to subsequent acute cellular rejection and ensuing allograft dysfunction [3, 4]. Not all clinically relevant tacrolimus neurotoxicity in posttransplant patients may necessitate a switch of the immunosuppressant. We present a case of tacrolimus-associated neurotoxicity in which psychiatric intervention led to clinical improvement without tacrolimus dose changes.

## 2. Case Report

A 45-year-old Hispanic male at 3 months after orthotopic liver transplantation was admitted to the liver transplant unit at an academic medical center with an index subacute onset of a manic episode with psychotic features. He experienced increased energy, decreased need for sleep, a habit of staying up all night on his computer, irritable mood, various delusions, and bizarre behavior. He became convinced of a plot by others in his community to harm him and his family members. He later became concerned that his family members were involved in the plot and accused them of the same. He believed that his dog was speaking to him about the plot and that he needed to “save” his family members. He expressed the belief that he had achieved the “powers of Jesus,” in that he had become clairvoyant to predict future events. Medical history included alcohol dependence in remission and use of multiple other drugs and cirrhosis attributed to NASH, alcoholism, and HCV. Medications included tacrolimus, 4 mg bid; mycophenolate, 1000 mg bid; prednisone, 5 mg daily; amlodipine, 10 mg daily; and metoprolol, 12.5 mg bid.

On psychiatric consultation, he was fully alert, distractible, restless, talkative, mildly expansive, jovial and joking, and circumstantial with only fair insight and judgment. He had a MMSE of 26/30. He denied suicidal/homicidal ideation, hallucinations, or delusions at the time of the evaluation. Tacrolimus trough level was 6.3 micrograms/liter (5.0–15.0). Urine drug screen was negative. Other laboratory studies were notable only for a slightly increased bilirubin. Liver and renal functions were unremarkable. CT of head was normal. Tacrolimus dosage was maintained. Treatment with olanzapine of 5 mg hs improved his sleep pattern and decreased his manic symptoms. Within 5 days, he has much improved. He was discharged on a dose of olanzapine of 5 mg hs in improved condition and he was referred to outpatient psychiatric follow-up.

## 3. Discussion

The emergence of mood, psychotic, and neurocognitive symptoms in a stable immunosuppressed posttransplant patient was interesting in the context of a therapeutic tacrolimus level. Tacrolimus neurotoxic symptoms range from psychiatric symptoms (e.g., anxiety, mood, and psychotic episodes) to neurological manifestations (e.g., tremors, dysarthria, apraxia, seizures, delirium, and coma), including a less frequent complication termed “posterior reversible encephalopathy syndrome” [1, 4–6].

Neuropsychiatric complications may also occur with other immunosuppressants; for example, mycophenolate can induce depression, and corticosteroids can precipitate anxiety, insomnia, mood, psychotic, and cognitive symptoms [7, 8]. However, the prominent adverse effects of mycophenolate appear to focus on a different profile, emphasizing gastrointestinal and hematologic effects [9]. In one study, switching to mycophenolate monotherapy was found to improve neurotoxicity associated with calcineurin inhibitors [9]. Corticosteroid-induced mood and cognitive symptoms often occur during the first few weeks of therapy [8]. While dose modification or discontinuation of the corticosteroid treatment may resolve some of these adverse side effects, psychotropic agents are often required because of the severity of the psychiatric symptoms. Notably, corticosteroids are inducers of the CYP3A4 enzyme involved in tacrolimus metabolism [10]. Thus, caution is necessary to monitor increasing tacrolimus levels (and, thus, overimmunosuppression) upon corticosteroid discontinuation. Whether this was a sole tacrolimus-related neuropsychiatric consequence or a cumulative effect of the triple immunosuppression therapy was impossible to determine with certitude. There were no electrolyte abnormalities, other medications, or medical conditions that could have explained his psychiatric presentation.

A lower threshold to switch tacrolimus to another agent has been the recommended current practice in the resolution of neurotoxicity, especially when mental status changes persist after transplantation [3]. However, switching tacrolimus can be impractical at times due to amplified risk of organ rejection [3, 4]. In our case, a prompt psychiatric treatment with olanzapine coupled with continued use of tacrolimus led to resolution of the manic episode without a need to modify the immunosuppressant therapy.

Careful monitoring for psychiatric symptoms during immunosuppressive therapy is required particularly for those with higher neurotoxic susceptibility. In liver transplant, age, model for end-stage liver disease (MELD) score, and organ functioning were found to predict posttransplant tacrolimus neurotoxicity [3]. Those with a pretransplant diagnosis of alcoholic cirrhosis were found to be at higher risk for neuropsychiatric symptoms [3]. But solely relying on tacrolimus concentrations to suspect or diagnose neurotoxicity can be misleading, as similar trough tacrolimus concentrations were found in those with and without neurotoxicity [1].

## 4. Conclusion

Immunosuppressant neurotoxicity may present with primarily neuropsychiatric symptoms, regardless of tacrolimus concentrations. In certain patients, continuing tacrolimus dosage, with close monitoring of serum concentrations, can be combined with psychiatric treatment with subsequent improvement in tacrolimus-associated psychopathology. Such cases may benefit from continued surveillance for psychiatric symptoms throughout the course of tacrolimus therapy.
